# The Inhibitory Effect of Niosome Containing Myrtenol as an Innovative Approach to Combat Methicillin-Resistant *Staphylococcus aureus* (MRSA)

**DOI:** 10.1155/cjid/2742569

**Published:** 2025-09-03

**Authors:** Jaber Hemmati, Mohsen Chiani, Babak Asghari, Ghodratollah Roshanaei, Sara Soleimani Asl, Morvarid Shafiei, Mohammad Reza Arabestani

**Affiliations:** ^1^Department of Microbiology, School of Medicine, Hamadan University of Medical Sciences, Hamadan, Iran; ^2^Department of Bacteriology, Pasteur Institute of Iran, Tehran, Iran; ^3^Department of NanoBiotechnology, Pasteur Institute of Iran, Tehran, Iran; ^4^Department of Biostatistics, School of Medicine, Arak University of Medical Sciences, Arak, Iran; ^5^Anatomy Department, School of Medicine, Hamadan University of Medical Sciences, Hamadan, Iran; ^6^Infectious Disease Research Center, Hamadan University of Medical Sciences, Hamadan, Iran

**Keywords:** biofilm, drug delivery system, methicillin-resistant *Staphylococcus aureus* (MRSA), myrtenol, niosome, resistant wound infection

## Abstract

The clinical challenge of staphylococcal treatment is increasing globally, making it critical to find effective strategies to hinder the spread of resistant isolates, particularly methicillin-resistant *Staphylococcus aureus* (MRSA). Niosomal drug delivery systems, known for their controlled release profiles and other advantageous features, can enhance the efficacy of antimicrobial ability of loaded agents. This study aims to propose a novel drug delivery system for combating staphylococcal resistance challenge through examining the antibacterial and antibiofilm properties of myrtenol-loaded niosomal system against MRSA isolates. The niosomal formulation was prepared using the thin-film hydration process, and its physicochemical characteristics were assessed through entrapment efficiency (EE %), in vitro release profile, field-emission scanning electron microscopy (FE-SEM), and dynamic light scattering (DLS). In addition, minimum inhibitory concentrations (MICs) and minimum bactericidal concentrations (MBCs) were measured and compared with free myrtenol to evaluate the anti-MRSA activity of the formulated niosomal myrtenol. Furthermore, the effectiveness of niosome containing myrtenol against MRSA biofilms was investigated by examining biofilm minimum inhibitory and eradication concentrations (BMIC/BMEC). Additionally, the cytotoxicity of synthesized niosomes was assessed on the human foreskin fibroblast cell line (HFF). Results from FE-SEM showed that myrtenol-loaded niosomes were spherical with a diameter of 122.1 nm, while the hydrodynamic size reported from DLS was 130.8 nm. The surface charge and EE% of the prepared niosomal formulation were −53.6 mV and 62.90%, respectively. The niosomal myrtenol formulation demonstrated the increased antibacterial activity in comparison with free myrtenol formulation. Furthermore, myrtenol-loaded niosomes reduced the biofilm formation potential in all MRSA isolates and effectively eradicated bacterial biofilms at equivalent concentrations of the non-niosomal formulation. According to this study, niosomes exhibit high potential for drug delivery due to several favorable characteristics, including a sustained-release profile, nontoxicity, small size, and high EE%. Niosomal delivery system presents a novel approach to combat bacterial infections, particularly those caused by MRSA isolates by enhancing antibacterial and antibiofilm activities of free myrtenol.

## 1. Introduction

*Staphylococcus aureus* is recognized as a major causative agent of nosocomial infections, as well as infections in diabetic and bedsores ulcers [1]. The indiscriminate use of methicillin as an effective antibiotic against *S. aureus* infections has led to the growing phenomenon of methicillin-resistant *S. aureus* (MRSA) strains, thereby causing significant therapeutic challenges in healthcare systems [2, 3]. MRSA strains are highly capable of forming biofilm, which is a substantial virulence factor in these strains [4] and provides bacterial shielding from unfavorable environmental conditions and immune responses [5]. In addition, biofilm can protect embedded bacteria against excessive concentrations of antimicrobial agents, causing resistant and complicated infections [6]. Furthermore, bacterial biofilm provides an ideal condition for transmitting genetic elements and spreading antibiotic resistance among the bacterial population [7]. On the other hand, water-insolubility [8], low absorption into targeted sites [8], in vivo instability [9], and dose-dependent side effects [10] are among the reasons causing the failure of conventional antibacterial agents against MRSA infections. Regarding the undeniable role of biofilm in MRSA pathogenicity and the inefficiency of conventional antibiotics, it seems necessary to find novel antibacterial and antibiofilm agents to overcome the therapeutic problems of this pathogen [11].

Alec Bangham, a British scientist, initially introduced vesicular drug delivery systems, which are composed of one or more concentric bilayer membranes [12]. These systems have a bilayer structure composed of amphiphilic molecules, which can be used for the encapsulation of lots of materials [13, 14]. Biocompatibility, biodegradability, simple formulation, and flexibility are the prominent advantages of these drug delivery systems, attracting significant interest from researchers [15, 16]. The copious lipid-base vesicular systems have been proposed as a new approach for different pharmaceutical purposes, particularly eradicating bacterial infections, in which niosomes are the most significant ones [17–19]. Since nonionic surfactants are a crucial ingredient in the formulation of niosomes, they are distinctly different from liposomes. This key component imparts desirable properties to niosomes, setting them apart in terms of stability and functionality [20]. Recently, various niosomal formulations have been developed to encapsulate antibacterial agents, offering significant advantages in combating bacterial infections [21, 22]. Among these agents, essential oils—naturally occurring hydrophobic liquids composed of diverse chemical classes (such as phenols, aldehydes, ketones, and alcohols)—have attracted attention due to their potent antibacterial properties [16].

Myrtenol, as a bioactive metabolite, constitutes the essential oils of various medicinal plants, including *Lippia multiflora* [23], *Myrtus communis* [24], *Rhodiola rosea* [24], *Turnera diffusa* [23], *Anemopsis californica* [23, 25], *Paeonia lactiflora*, and *Tanacetum vulgare* [23, 24]. This fragrance component is often utilized in a wide range of cosmetic products and has been recognized as a safe flavoring ingredient by the European Food Safety Authority (EFSA) [26]. In traditional medicine, the application of myrtenol for treating gastrointestinal pain, anxiety, inflammation, and bacterial infection has been explored [27]. Besides nontoxicity, various biological activities of myrtenol, including anticancer [28], anti-Alzheimer's [29], antioxidant [30], antimicrobial [23], and antibiofilm [23], were also approved. Notably, it has been found that myrtenol has inhibitory effects on MRSA virulence factors and biofilms, which could have a wide administration for eradicating infections related to this superbug [23].

Because of their hydrophobic nature, essential oils can integrate into the cytoplasmic membrane, causing leakage of cellular components and ultimately leading to bacterial cell death. However, these oils tend to be volatile and unstable [16]. When encapsulated within niosomal nanocarriers, the stability of essential oils is enhanced by enabling their controlled and sustained release [31, 32]. Numerous studies have indicated that niosomes have incredible potential against due to effective delivery in infected or injured sites [21, 22, 33–35]. In this study, myrtenol-encapsulated niosomes were formulated and their morphological characteristics, release profile, toxicity effect, and entrapment efficiency (EE %) were examined. Also, we aim to suggest an effective and novel anti-MRSA agent through evaluating the antibacterial and antibiofilm activities of myrtenol-loaded niosomes.

## 2. Materials and Methods

### 2.1. Materials

Span 60, Tween 60, methanol, crystal violet, chloroform, glycerol, all culture mediums, and Amicon ultracentrifugal filter (MWCO 50 kDa) were purchased from Merck Company, Germany. Myrtenol was purchased from Sigma-Aldrich (India) and dissolved in methanol to prepare a stock solution at a final concentration of 10 mg/mL for each experiment. The dialysis membrane (MWCO 12 kDa) known as Spectra/Por was also obtained from Sigma-Aldrich, USA.

### 2.2. Bacterial Isolation

In this study, 12 MRSA strains were collected from human clinical wounds, including diabetic ulcers and bedsores, using sterile cotton swabs, and were used for antimicrobial analysis [36]. The biofilm-forming potential of MRSA strains was approved using the microtiter plate method [36]. The isolates were stored in trypticase soy broth (TSB) medium with 15% glycerol at −20°C for further analysis [36]. Also*, S. aureus* (MRSA) ATCC 6538 donated from microbial collection bank of Pasteur Institute of Iran was used as a standard strain.

### 2.3. Preparation of Myrtenol-Loaded Niosomes

In the current work, the thin-film hydration procedure was employed in the preparation of niosomal formulation [37]. Initially, specific amounts of Span 60, Tween 60, cholesterol, and myrtenol with a molar ratio of 2:2:1:0.6 (188.4 mg: 169.2 mg: 287.0 mg: 20.0 mg) were dissolved in 20 mL of organic solvent (2:1 v/v of chloroform–methanol solution) using a magnetic stirrer (150 rpm, 50 min, 25°C) to obtain a homogenous suspension. The organic solvent was then evaporated under vacuum at 60°C for 50 min at 150 rpm using rotary evaporation (WB Eco Laborota 4000 Model, Heidolph, Germany). By purging the nitrogen gas for 10 min, the residual solvent was removed from solution. The dried lipid was then hydrated for 45 min in 20 mL of sterile saline solution (PBS, pH 7.2, 5 mM). Finally, a 10-min sonication (Hielscher up 50H ultrasonic processor, Germany) was applied to the niosomal formulation [38, 39]. Following the same protocol, the blank niosomal formulation was synthesized without adding of myrtenol. Finally, the prepared formulations were visually examined for turbidity and flocculation and kept for further experiment.

Notably, the unentrapped drug was separated from niosomal myrtenol formulation, and an equal amount of free myrtenol was compared to the encapsulated drug in all experiments.

### 2.4. Characterization of Niosomal Physicochemical Features

#### 2.4.1. Evaluation of Particle Hydrodynamic Size and Zeta Potential

Hydrodynamic size, polydispersity index (PDI), and surface zeta potential of prepared formulation were analyzed by dynamic light scattering (DLS) method using a zeta-sizer instrument (Malvern Instruments, UK) at 633 nm. The samples were tested in a polystyrene cuvette at the same temperature, concentration, and pH (25°C, 0.1 mg mL^−1^, 7.4) [22]. The results were analyzed three times and reported as mean ± SD, and PBS buffer was considered as the control sample in DLS method.

#### 2.4.2. Particle Morphology

Field-emission scanning electron microscopy (FE-SEM, Hitachi, S4160, Japan) was utilized in this study to assess the niosomes' size and morphology. To do FE-SEM imaging, one drop of a 1:100 diluted nanoparticle suspension in deionized water was placed on a plate and covered with a coating of conductive gold. Finally, the images captured during FE-SEM analysis were examined using ImageJ software, which was included with Java 1.8.0_172 [22, 40].

#### 2.4.3. Determination of EE%

The EE% of niosomal myrtenol formulation was investigated by the ultrafiltration protocol. Briefly, 1 mL of drug-encapsulated niosomal formulation was centrifuged in an Amicon ultra centrifugal filter at 14,000 g for 15 min at 4°C. By using a standard curve and UV spectrophotometry (Jasco V-530, Japan) at 225 nm, the amount of myrtenol in the supernatant solution was determined. The following formula was used to calculate EE% of myrtenol in niosomal formulation [14, 41]:

(1) $$\begin{array}{c} \text{EE}\%=\frac{\text{amount of initial loaded myrtenol}-\text{amount of myrtenol in supernatant}}{\text{amount of initial loaded myrtenol }}\times 100. \end{array}$$

#### 2.4.4. In Vitro Release Study

Using a dialysis technique, the in vitro myrtenol release from niosomal formulation was examined. After separating the unentrapped myrtenol, 1 mL of noisome containing myrtenol was placed in dialysis bag. Afterward, the dialysis bag was submerged in 25 mL of PBS (pH = 7.4, 5 mM) as the recipient medium, and it was magnetically stirred at 37°C at 100 rpm. At the subsequent stage, 1 mL aliquots of the receptor medium were aliquoted at 1, 2, 4, 8, 24, and 48 h intervals, and the absorbance of each sample was measured spectrophotometrically at 225 nm. Finally, the released drug concentrations at each interval time were assessed according to the standard curve equation. To maintain sink conditions and ensure accurate measurement of cumulative drug release, the recipient medium was completely replaced at each interval time [19].

Notably, same amount of free myrtenol was compared with the encapsulated drug in the release study.

#### 2.4.5. Stability Studies

To monitor the prepared formulation stability, the drug EE%, hydrodynamic size, and PDI of the myrtenol-loaded niosomes were evaluated every month for 4-month storage at 4°C and 25°C [14, 19, 32, 42].

#### 2.4.6. Cytotoxicity Determination of Niosomal Myrtenol

The produced niosomes' cytotoxicity was assessed using (dimethylthiazol-2-yl)-2,5-diphenyl-tetrazolium bromide (MTT) technique on human foreskin fibroblast cell line (HFF, supplied from National Cellular Bank of Pasteur Institute, Iran). In summary, 200 μL of RPMI-1640 media was used to seed 10^5^ cells per well into a 96-well microtiter plate. Afterward, the increasing concentrations of free and entrapped myrtenol were exposed to cells for 24 h. Then, the wells were resuspended in 15 μL of 5 mg mL^−1^ MTT solution and the plate was incubated for 4 h at 37°C. To solubilize the formazan purple crystals, 100 μL of dimethyl sulfoxide (DMSO) was added to the plate. In the final step, the absorbance of each well was measured at 570 nm using a microplate reader (AccuReader, Taiwan) [43].

### 2.5. In Vitro Assessment of Antibacterial and Antibiofilm Activities

#### 2.5.1. Minimum Inhibitory/Bactericidal Concentration (MIC/MBC)

MICs of myrtenol and myrtenol-loaded niosomes against MRSA clinical strains were evaluated using the approved CLSI broth microdilution method [44]. For this purpose, 96-well microdilution plates were filled with Mueller-Hinton broth (MHB), along with serial dilutions of free and niosomal formulations. Following the addition of 0.5 McFarland suspensions of bacteria to each well, the plates were incubated at 37°C overnight. MICs reported the lowest concentrations causing nonobservable bacterial growth after overnight incubation. Also, MBCs were evaluated as the lowest dilution leading to no growth (> 99%) after overnight incubation on Mueller–Hinton agar (MHA). MRSA ATCC 6538 and uninoculated mediums were utilized as positive and negative controls, respectively. It should be mentioned that all assays were carried out in triplicate.

#### 2.5.2. Biofilm Minimum Inhibitory and Eradication Concentration (BMIC/BMEC)

96-Well microtiter sterile plates were seeded with 200 μL of diluted bacterial suspension (10^6^ CFU/mL) with TSB medium supplemented with 1% glucose in order to assess the BMIC of niosomal myrtenol and compare with the free drug. Following the addition of serial dilutions of free and niosomal formulations, the microplates were incubated overnight at 37°C. The wells were then washed three times with sterile PBS (pH = 7.4, 5 mM). Ultimately, the biofilms underwent fixation and staining using 99.8% methanol and 1.5% w/v crystal violet solution, respectively. The microplate reader was used to record the ODs of the wells in triplicate at 570 nm. Based on the following criteria, the BMICs were defined as the lowest dose that caused at least 90% suppression in biofilm formation in comparison to untreated biofilm [45–47]:

(2) $$\begin{array}{c} \text{Biofilm inhibition }\%=\frac{\left(\text{the OD of untreated biofilm}\right)-\left(\text{the OD of treated biofilm}\right)}{\left(\text{the OD of untreated biofilm}\right)}\times 100. \end{array}$$

BMECs were performed to determine the efficacy of myrtenol-containing niosomes compared to free drug against preformed bacterial biofilms. In summary, the isolates were allowed to form 1- and 3-day-old biofilms, as mentioned above. After washing biofilms with sterile PBS (pH = 7.4, 5 mM), the wells were then treated with serial dilutions of free and niosomal formulations. Following an overnight incubation period at 37°C, a 10 μL aliquot from each treated well was carefully collected and spread onto MHA plates. These plates were then incubated for 48 h at 37°C to allow any surviving bacteria to grow. The lowest concentration eradicating all of the implanted bacteria was determined by counting the number of MRSA colonies which was reported as the BMEC value [48].

### 2.6. Statistical Analysis

ANOVA and *t*-test were used as the statistical test to assess investigated parameters. Also, the significant level was considered at *p* value < 0.05 for all tests. All graphs were designed using GraphPad Prism Version 9.0 software.

## 3. Results

### 3.1. Characterization of Myrtenol-Loaded Niosomes

#### 3.1.1. Determining Hydrodynamic Size, Zeta Potential, and PDI

**Figure 1:**
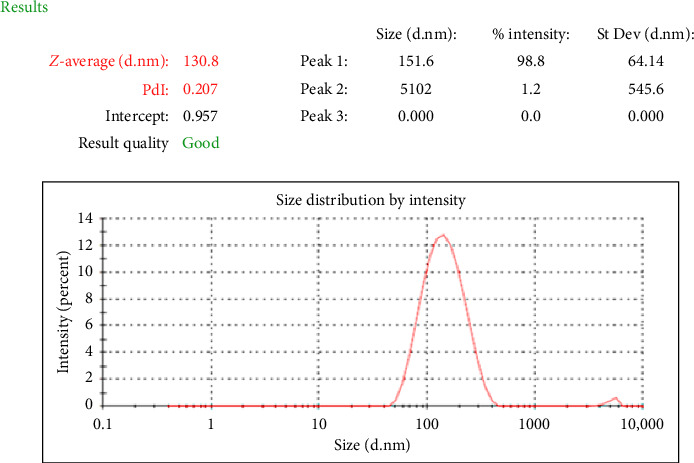
Size distribution curve of niosomes containing myrtenol using dynamic light scattering (DLS) method.

The hydrodynamic diameter measured by the zeta-sizer was 130.8 ± 12.5 nm, and the size distribution (PDI) of niosome nanoparticles was reported to be 0.207 ± 0.011, indicating homogenous dispersion of niosomes (Figure 1). Additionally, the surface charge of niosome containing myrtenol was reported to be −53.6 ± 1.6 mV.

#### 3.1.2. Particle Morphology

**Figure 2:**
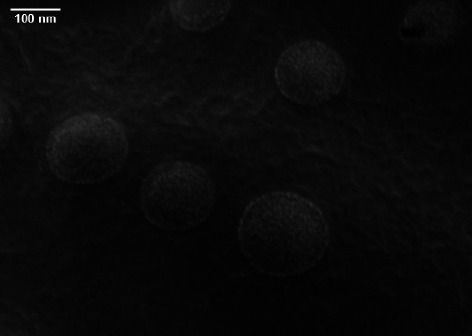
Morphology and size determination of niosomes containing myrtenol based on the field-emission scanning electron microscopy (FE-SEM).

Based on the micrograph obtained from FE-SEM, the myrtenol-loaded niosomes were uniform spherically shaped and had almost identical size with 122.1 nm diameter (Figure 2).

#### 3.1.3. Niosomal EE%

Our findings showed that the amount of incorporated drug in niosomes was 62.90%. Considering the synthesized niosomes' size, the reported EE% was notably high, indicating effective encapsulation of myrtenol within the niosomal nanocarriers [29, 31].

#### 3.1.4. In Vitro Release

**Figure 3:**
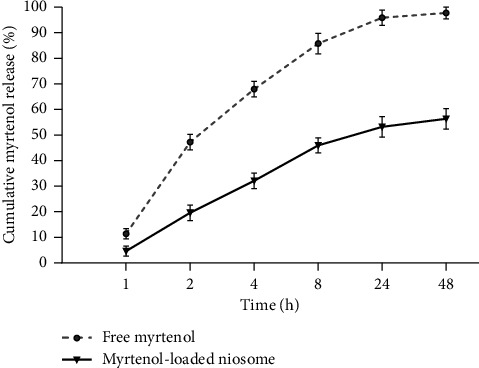
Comparison of release profiles between free myrtenol and myrtenol-loaded noisome.

Figure 3 shows the myrtenol release profiles from the dialysis bag, incorporating the free and niosomal formulations. It can be seen that in the first 4 hours, around 68% of the non-niosomal formulation of myrtenol was released from the dialysis bag, whereas only 32% of the loaded myrtenol was released from the niosomal carriers at the same time. Similarly, the maximum rate of drug release in the niosomal formulation was 56.3% during 48 h, approximately 100% of the drug was released from free myrtenol formulation. Comparative release of free and niosomal formulations showed the possibility of inhibition of the massive drug release by loading myrtenol in niosomes.

#### 3.1.5. Physical Stability

**Figure 4:**
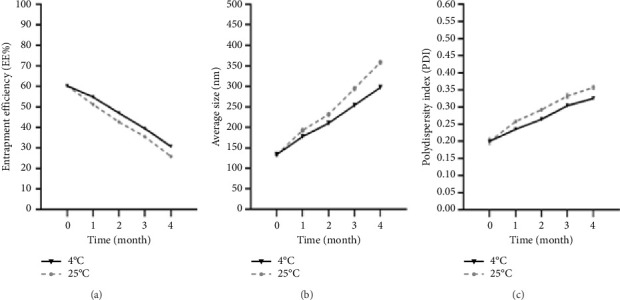
The stability study of niosomal formulation stored at two temperature conditions (25°C and 4°C) during 4 months. (a) Entrapment efficiency, (b) hydrodynamic size, and (c) polydispersity index (PDI).

The physical stability of synthesized myrtenol-loaded niosome was examined via determining PDI, EE%, and hydrodynamic size during a 4-month storage at two temperature conditions (25°C and 4°C) (Figure 4). The results indicated that the changes in all investigated parameters at 4°C were lower than 25°C. Notably, the statistically significant difference in EE%, hydrodynamic size, and PDI was not observed between both storage conditions (*p* value > 0.05).

#### 3.1.6. Niosome Cytotoxicity

**Figure 5:**
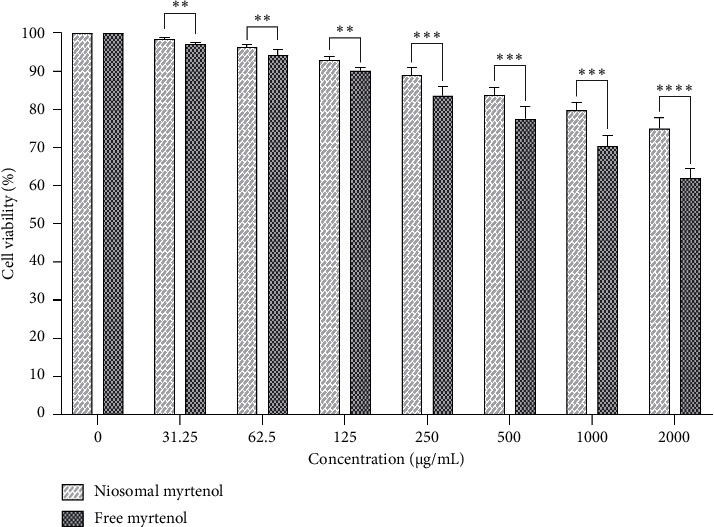
Comparison of HFF cell viability between free myrtenol and niosomal formulation after 24 h (mean ± SD, *n* = 3, ^∗∗^*p* value < 0.01, ^∗∗∗^*p* value < 0.001, ^∗∗∗∗^*p* value < 0.0001).

In our study, the cell viability of myrtenol-loaded niosomes and free myrtenol at various doses was assessed on HFF cells after 24 h. As shown in Figure 5, both free and niosomal myrtenol reduced cell viability in a dose-dependent manner. At each tested concentration, cell viability was significantly higher in the niosomal myrtenol group compared to the free myrtenol group. Statistical analysis revealed that these differences were significant as follows: *p* value < 0.01 at 31.25, 62.5, and 125 μg/mL; *p* value < 0.001 at 250, 500, and 1000 μg/mL; and *p* value < 0.0001 at 2000 μg/mL. These results demonstrate that encapsulation of myrtenol in niosomes reduces its cytotoxicity relative to the free form, especially at higher concentrations. Furthermore, the cytotoxicity of the blank formulation was evaluated and no discernible toxicity against HFF cells was found after 24 h.

### 3.2. Antibacterial Ability of Formulated Niosome

**Figure 6:**
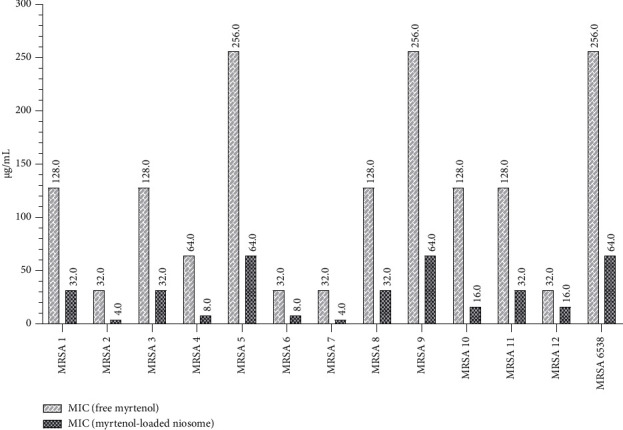
Comparison of the inhibitory effects of free myrtenol and niosomal formulation against MRSA clinical isolates.

**Figure 7:**
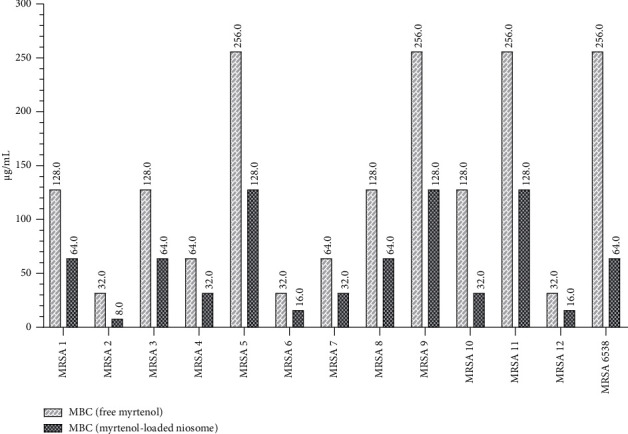
Comparison of the bactericidal effects of free myrtenol and niosomal formulation against MRSA clinical isolates.

The values of MIC and MBC of the niosomal myrtenol formulation were assessed and compared to that of the free drug against MRSA clinical isolates (Figures 6 and 7). Our results demonstrated that incorporation into niosomes reduced MICs of the free drug by 2- to 8-fold against all MRSA isolates. Furthermore, the prepared niosomes significantly enhanced the inhibitory activity of free myrtenol (*p* value < 0.01). Additionally, the findings indicated that niosomal encapsulation significantly improved the bactericidal efficacy of the free drug (*p* value < 0.05), with MBCs reduced by 2- to 4-fold in all isolates compared to free myrtenol. Notably, the antibacterial efficacy of blank niosome formulation was also assessed, which lacked antibacterial activity against all isolates.

### 3.3. Antibiofilm Activity of Formulated Noisome

The antibiofilm abilities of niosome containing myrtenol against MRSA strains were analyzed by determining the BMIC and BMEC rates and compared with the free form (Table 1). For BMIC determination, free and niosomal formulations were added to planktonic bacteria, whereas in BMEC assay, 1-day and 3-day-old biofilms were treated with the drugs. Stock solutions of both free and niosomal formulations were serially diluted in culture medium to obtain concentrations ranging from 1024 to 2 μg/mL. The volume of the added formulations was carefully adjusted to maintain a constant total culture volume in each well, thereby preventing dilution effects. According to our results, the niosomal formulation decreased BMIC values by 2–8-fold in all isolates compared to free myrtenol, and significantly inhibited biofilm formation (*p* value < 0.01) was found. Furthermore, the findings of the BMEC assay indicated that encapsulated myrtenol eradicated 1- and 3-day-old MRSA biofilms at lower concentrations compared to the free myrtenol formulation. Furthermore, the antibiofilm efficacy of blank niosomes was investigated, which had no antibiofilm effect at the same concentration of niosomal myrtenol formulation.

## 4. Discussion

MRSA is known as the seriously life-threatening pathogen causing high rates of mortality and morbidity. Recently, drug resistance to MRSA is rapidly expanding worldwide [49], and conventional antibiotics have challenged the treatment of MRSA infections [50]. Developing a new approach to eradicate resistant staphylococcal infections, especially those associated with MRSA strains, is urgently indispensable. Our study indicated that the anti-MRSA activities of niosome containing myrtenol were investigated, and the potential of niosomal drug delivery system was proven. In our research, MICs and MBCs results showed the potential of niosomal myrtenol as an effective antibacterial delivery system. Furthermore, numerous experiments reported that niosomal encapsulation improved the antibacterial activity of other natural compounds such as berberine [51], thymol [52], propolis [53], curcumin [34, 35, 54], carvacrol [33], and others. The enhanced antibacterial activity of encapsulated drugs may be due to the interaction between niosomal nanocarriers and bacteria [55, 56]. The niosomal vesicular system can undergo interaction with bacterial cell walls, resulting in increasing the permeability of the content released into subcellular space [56, 57]. The exact mechanism of niosome–cell wall interaction has been attributed to niosomal bilayer fluidity, inducing intracellular drug release by creating a concentration gradient [58]. Therefore, niosomal encapsulation through enhancing drug-effective doses of loaded contents could be applied for effective drug delivery against MRSA infections.

The MRSA isolates showed a high capability for biofilm formation and had a greater tendency to produce biofilms compared to those that were susceptible to methicillin [59]. The effective management of MRSA biofilm-associated complications relies on inhibiting bacterial attachment and development of biofilms, where the bacteria embedded in the biofilm exhibit higher drug resistance compared to planktonic cells [60]. In the present research, the inhibitory ability of niosomal entrapment on MRSA biofilm was evaluated, and it was exhibited that niosomal formulation significantly reduced the BMIC values of free drug in all tested isolates (*p* value < 0.01). Also, Barakat et al.'s [61] study results indicated that niosomal antibiotics had a lower BMIC for MRSA strains in comparison with free form. Additionally, in the confirmatory study by Dwivedi et al. [62], they proved the antibiofilm activity of niosomal encapsulation, where the adhesion of *S. aureus* isolates to the abiotic surface was greatly reduced by coating them with the encapsulated drug. Accordingly, niosomes could inhibit biofilm formation due to the facilitation of their diffusion into the biofilm matrix and efficient drug delivery into the embedded bacteria. Additionally, the persistent accessibility of drugs could be increased by controlling release from niosomes, which prevents resistance mechanisms in biofilm-forming bacteria [34, 63]. Furthermore, biofilm-forming bacteria can compete with niosomes as a physical barrier for surface attachment, which essentially means that niosomes can hinder the formation of biofilms by lowering bacterial adherence [64–66]. So, concerning the MRSA isolates' high potential in surface adhesion, niosomal drug delivery could be further developed for solving the therapeutic challenges related to MRSA biofilm.

Because biofilm is known as a significant MRSA virulence factor, eradicating formed biofilm on different abiotic and biotic surfaces becomes a critical issue for health care systems [67]. Our findings showed the significant eradication ability of the prepared formulation against MRSA biofilms, where myrtenol-loaded niosome reduced in BMEC values of free drug. The effect of niosomes on biofilm destruction was approved in another study, and cefazolin-encapsulated niosomes showed a higher biofilm eradication rate than free drug [68]. Also, Kashef et al.'s study confirmed the efficacy of niosomal encapsulation against MRSA biofilm, and it was determined that ciprofloxacin-loaded niosomes greatly decreased the density of the formed biofilm at the same concentration of free ciprofloxacin. It can be inferred that niosomes, through increasing the drug transfer into embedded bacterial cells, can enhance the effective dosage of loaded drugs, leading to fewer drug usage and intake intervals against chronic MRSA infections [16, 32].

Niosomes are composed of nonionic surfactants, providing favorable features in drug delivery. Nanoparticle size measurement is a key parameter that influences the stability and efficacy of niosomal formulations. Accurate characterization of nanoparticle size is crucial for understanding their behavior and performance in drug delivery systems [69]. Our study revealed that the nanoparticles' size reported by the nano zeta-sizer was larger than that obtained by FE-SEM. These contradictory results might be due to the difference in size determination between DLS and SEM methods. In other words, the measurement of DLS happens through the hydrodynamic diameter of niosomes, involving the core and all attached molecules on the surface, including ions and water. In contrast, SEM reports the niosomes diameter in a dried form and determines the exact size of each nanoparticle [50]. Therefore, combining complementary techniques like DLS and SEM provides a more comprehensive understanding of nanoparticle size and morphology, which is essential for optimizing the stability and therapeutic efficacy of niosomal drug delivery systems.

The presence of cholesterol in the niosomal structure has positive effect on drug EE%, permeability, and flexibility [70]. Also, the surfactant/cholesterol molar ratio is considered an important factor in enhancing niosomal EE%. Compared to our niosomes' size, the percentage of encapsulated myrtenol in niosomal dispersion was high (62.9%), resulting from its formulation containing a 4:1 molar ratio of surfactant/cholesterol [29, 31]. Zafari et al. [68] also prepared a niosomal formulation in a 7:3 molar ratio of Span 60/cholesterol that obtained 95.7% EE for cefazolin. Also, Akbari et al.'s research [71] demonstrated that the highest EE% was related to that formulation prepared with a 3:4 molar ratio of Spans 60 to cholesterol. Moreover, another study's findings [40] revealed that increasing the surfactant/cholesterol ratio improves the EE% of niosomal formulations. However, some studies [72, 73] reported contradictory results, where an increase in cholesterol amount declined the EE%. This decreasing effect showed that the high cholesterol level could disrupt the niosomal bilayer membrane, resulting in leakage of loaded contents [74]. Therefore, one of the significant factors in achieving the highest EE% in niosomal formulations was found to be the surfactants' balance to cholesterol mixture.

The electrical neutrality of nonionic surfactants leads to more stability in niosomal formulations, which could enhance their pharmaceutical behavior [75]. It is proven that the length of surfactants is a significant factor affecting the stability of niosomes during storage time. In our study, Span 60 and Tween 60 were used for preparing niosomal formulation, and their stability (EE%, hydrodynamic size, PDI) was investigated at the storage time of 4°months. The stored niosomes' stability at 4°C was more than that kept at 25°C, which is possibly due to the lower temperature causing higher rigidity of the hydrophobic ingredient of niosome. The findings of other studies by Manosroi et al. [76] and Junyaprasert et al. [77] indicated that vesicles synthesized with the long-chain surfactants (Span 60 and Tween 60) had more stability than those synthesized with the shorter-chain surfactants (Span 40 and Tween 40). However, increasing the length of incorporated surfactants could cause other effects on niosomes' properties, including bilayer thickness, hydrophobic moiety, membrane rigidity, and others [78]. Therefore, choosing appropriate surfactants must be considered for optimizing niosomal formulation based on different administration purposes.

## 5. Conclusion

This study demonstrates that myrtenol-loaded niosomes exhibit significant antibacterial and antibiofilm effects against MRSA isolates. Importantly, our findings highlight the potential of niosomal myrtenol as a promising therapeutic candidate for combating MRSA infections, particularly those involving biofilm-associated resistance. Encapsulation of myrtenol in niosomal delivery system not only enhances its pharmacological profile but also addresses the urgent need for alternative strategies to overcome resistant infections caused by MRSA isolates. These results support further investigation of niosomal myrtenol as an innovative antibiofilm and antibacterial agent in clinical settings.

## Figures and Tables

**Table 1:** Biofilm minimum inhibitory and eradication concentrations (BMICs and BMECs) of free and niosomal formulations against MRSA clinical isolates.

MRSA isolates	BMIC (μg/mL)		BMEC (μg/mL)			
	Free myrtenol	Niosomal myrtenol	Free myrtenol		Niosomal myrtenol	
			1-day biofilm	3-day biofilm	1-day biofilm	3-day biofilm
Iso. 1	256	64	512	1024	128	512
Iso. 2	64	16	256	512	128	256
Iso. 3	512	128	1024	> 1024	512	1024
Iso. 4	128	16	512	1024	128	512
Iso. 5	512	256	> 1024	> 1024	1024	> 1024
Iso. 6	64	8	256	512	64	256
Iso. 7	128	32	512	1024	128	512
Iso. 8	256	64	512	> 1024	256	1024
Iso. 9	512	256	> 1024	> 1024	1024	> 1024
Iso. 10	256	64	512	> 1024	256	1024
Iso. 11	512	256	1024	> 1024	512	1024
Iso. 12	64	32	256	512	64	128
MRSA 6538	512	256	> 1024	> 1024	1024	> 1024

## Data Availability

Data are available upon request from the authors.
